# Modeling Validation and Control Analysis for Controlled Temperature and Humidity of Air Conditioning System

**DOI:** 10.1155/2014/903032

**Published:** 2014-08-26

**Authors:** Jing-Nang Lee, Tsung-Min Lin, Chien-Chih Chen

**Affiliations:** ^1^Department of Refrigeration, Air-Conditioning and Energy Engineering, National Chin-Yi University of Technology, Taichung 411, Taiwan; ^2^Graduate Institute of Mechanical and Electrical Engineering, National Taipei University of Technology, Taipei 106, Taiwan

## Abstract

This study constructs an energy based model of thermal system for controlled temperature and humidity air conditioning system, and introduces the influence of the mass flow rate, heater and humidifier for proposed control criteria to achieve the controlled temperature and humidity of air conditioning system. Then, the reliability of proposed thermal system model is established by both MATLAB dynamic simulation and the literature validation. Finally, the PID control strategy is applied for controlling the air mass flow rate, humidifying capacity, and heating, capacity. The simulation results show that the temperature and humidity are stable at 541 sec, the disturbance of temperature is only 0.14°C, 0006 kg_w_/kg_da_ in steady-state error of humidity ratio, and the error rate is only 7.5%. The results prove that the proposed system is an effective controlled temperature and humidity of an air conditioning system.

## 1. Introduction

The air conditioning system can regulate indoor air quality to provide a more comfortable and safer environment. It is also important in the high-tech industry and medical industry. Liu et al. [1] use computational fluid dynamics (CFD) to create a thermally comfortable and healthy environment by air distributed information from numerical simulation. Safaei and Goshayshi [2] finite volume method, which is based on the difference of temperature and momentum, has effect on mechanical ventilation or pressure different and finally results in indoor and outdoor flow. Safaei et al. [3] investigate the energy transferring through two different heated side walls, especially transferring in rooms and buildings. However, as the controller of air-conditioning system has long time constant, multivariable, and high control complexity by interaction between parameters, it is thus difficult to obtain accurate mathematical model. Due to the high energy consuming, the research on high efficiency energy-saving air conditioning system has become a topical subject. An effective control law is very important for developing high energy efficiency air-conditioning system. The discussion on the influence of air-conditioned zone energy input and output on indoor temperature and humidity, as well as the analysis of the dynamic characteristics of air conditioning system, is important in analyzing air conditioning system and designing control law. Therefore, it is necessary to develop an approximate thermodynamic model and validate the feasibility of model at the design stage of air conditioning system. Many recent studies have used the theoretical or experimental method to explore the dynamic model of air conditioning system as the basis of air conditioning system control. Tsao et al. [4] developed high efficiency make-up air units (MAUs) for technology factories in subtropical zone by modeling thermodynamic system and proposed eight schemes for analysis, among which was the dry coil cooling- (DCC-) based energy-saving scheme. However, the thermal model was built for air-conditioning equipment only, and the effect of energy on the state of air-conditioning space was not discussed. Soyguder et al. [5] built a thermal model of air conditioning system and used PID expert fuzzy control to adjust the air-conditioned zone temperature change. However, that system lacked humidity control for air-conditioned zone. Tashtoush et al. [6] developed a thermal model of air conditioning system and air-conditioned zone and used PID to control the outflow air temperature and humidity for constant temperature and humidity control. However, only the outflow air temperature and humidity were controlled, while the operation of the equipment was not discussed. The outflow air temperature and humidity matching problem of the chilled water was not considered. Rehrl and Horn [7] proposed a simple thermodynamic model of air-conditioning equipment and combined it with feedback method for predictive control of air conditioning system. However, in terms of humidifier, the control mode was designed according to the characteristic curve of valve and humidifying capacity, and the test room was built instead of thermal model of air-conditioned zone for control. Platt et al. [8] built a model of air conditioning system and used genetic algorithm to optimize air conditioning control. However, only the temperature change of air-conditioned zone was controlled and the thermal model of air-conditioning equipment was not built, and the genetic algorithm was used as control base. Yao et al. [9] predicted the variance in chilled water by building the thermal model of chilled water coil and validated it by simulation. However, they did not apply the control law to the air-conditioned zone. Soyguder and Alli [10] used the air conditioning system thermal model and applied input-output data to PID ANFIS to control the fan speed. Barbosa and Mendes [11] built the thermal model of various equipment types of central air conditioning system and used weather data to simulate the on-off control of chiller. Kasahara et al. [12] modeled the air-conditioned zone and controlled temperature and humidity air conditioning system and used the thermal model and PI control to change the air output for constant temperature and humidity. Riederer et al. [13] built the thermal model of room system but did not regard the room as uniformly mixed constant volume. The air temperature change was deduced from the positions of sensors, and it was validated by experimental simulation. Kang et al. [14] proposed the HVAC model which introduces CO_2_ concentration to consider the comfort of room, and linear-quadratic regulator (LQR) is applied for optimizing and stabilizing the system. Amos-Abanyie et al. [15] investigated the thermal mass effect, window size, and night ventilation on peak indoor air temperature (PIAT) via E plus (E+) simulation. Homod [16] analyzed the advantage and disadvantage from types of HVAC model.

Therefore, in order to implement constant temperature and humidity control, the controlled temperature and humidity air conditioning system was used in this study. The air feeder, cooling coil heater, and humidifier constituted the air-conditioned zone at constant temperature and humidity. The thermal model of controlled temperature and humidity air conditioning system and air-conditioning equipment was built to discuss the influence of various types of equipment in the air conditioning cabinet on the temperature and humidity of air-conditioned zone. Finally, considering the matched cases, the PID control law was used to control the air mass flow rate (${\dot{m}}_{a}$), heater input percentage (*α*), and humidifier input percentage (*γ*). This study investigates the controlling based on thermal and humidity conditions only without considering the energy transferring such as CFD, thus to discuss the stabilization time, overshoot and stability of controlled temperature, and humidity air conditioning system under PID control.

## 2. Modelling

### 2.1. Description

**Figure 1:**
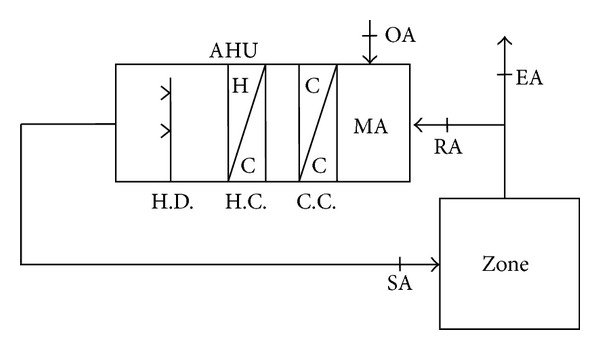
Controlled temperature and humidity of air conditioning system thermal model diagram.

The main function of controlled temperature and humidity air conditioning system is to make the air-conditioned zone at constant temperature and humidity by applying different control strategies, so as to meet different industrial requirements, such as chemical laboratory, measuring and testing laboratory, process of high-tech industry, and electronic machine room.  Figure 1 shows a typical controlled temperature and humidity air conditioning system.

First the difference between the practical status and target value is obtained by detection of indoor air state. Then the flow of return air (RA) is controlled by exhaust air (EA) and the outdoor air (OA) is introduced to generate mixing air (MA) in air-handling unit. The mixing air is processed by cooling and dehumidifying by cooling coil (C.C.), heating by heating coil (H.C.), and humidifying by humidifier (H.D.) to mix the desired state for air-conditioned zone gradually.

**Figure 2:**
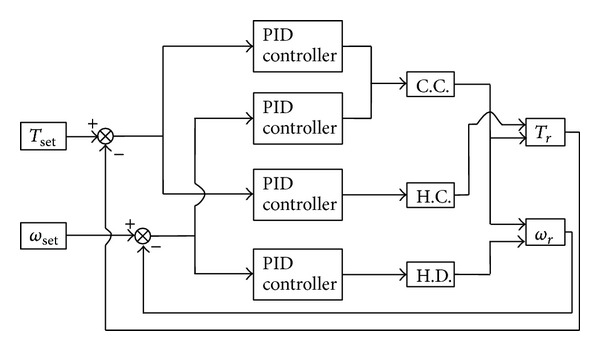
Overall block diagram for controlling the proposed air conditioning system.

As shown in Figure 2, the algorithm first compares the difference between given *T*_*s*_, *ω*_*s*_ and room *T*_*r*_, *ω*_*s*_, respectively, and delivery to PID controller. The control parameters *m*_*a*_, *α*, and *γ* of C.C, H.C., and H.D. are changed and begin to affect the room temperature and humidity. The controlled *T*_*s*_, *ω*_*s*_ are compared again with room *T*_*r*_, *ω*_*s*_ and begin the next loop.

### 2.2. Mathematical Model of Controlled Temperature and Humidity of Air Conditioning System

Figure 1 shows that, under the control of chilled water coil, heater, and humidifier of the controlled temperature and humidity air conditioning system, the air-conditioned zone is provided with appropriate energy to keep its temperature and humidity corresponding to the set conditions. The modeling of the system of the exhaust air, mixing air, chilled water coil, heater, and humidifier components is discussed. The design of comprehensive mathematical model of a controlled temperature and humidity air conditioning system is unpractical and unreasonable due to the complexity from its multivariable. Therefore the mathematical model will be simplified by the following assumptions.

1. The specific heat and density of air are constants.
2. There is no air leakage in the process.
3. The boundary is insulated.
4. The air is regarded as the ideal gas.

#### 2.2.1. Exhaust Air and Mixing Air Model

**Figure 3:**
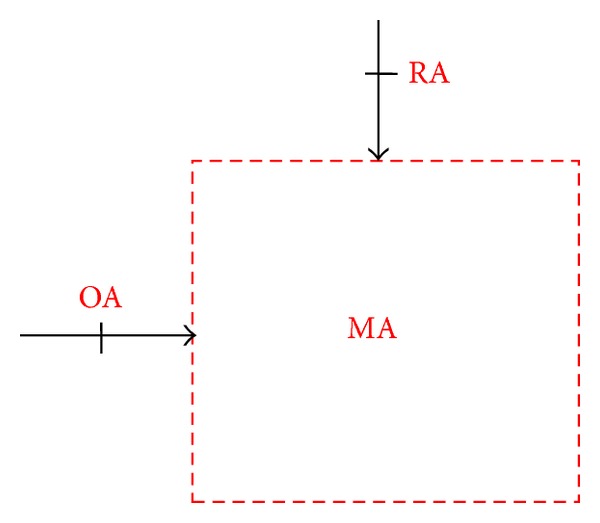
Schematic controlled volume of mixing process.

Generally, introduction of the outside air conditioning will raise cooling load on the air-conditioning zone. However, an appropriate amount of outside air can dilute the hazardous material in the air-conditioned zone, thus providing necessary fresh air for breathing, removing pollutant and controlling the temperature and humidity of work site, and upgrading the indoor air quality. The exhaust air damper is linked to outside air damper and controls the air discharge and outside air volume.  Figure 3 shows the mixing process of indoor return air and outside air in the air mixing zone of the air conditioning cabinet, the outside air and return air volumes are adjusted by controlling the outside air damper opening, and the air is mixed in the mixing zone of air conditioning cabinet.

If the mixing process is a steady flow process, the energy conservation for the mixing process is expressed as

(1) $$\begin{array}{c} \left(1-\beta \right)\times {\dot{m}}_{a}c_{pa}T_{\text{RA}}+\beta \times {\dot{m}}_{a}c_{pa}T_{\text{OA}}={\dot{m}}_{a}c_{pa}T_{\text{MA}}\\ \beta \times {\dot{m}}_{a}c_{pa}\left(T_{\text{OA}}-T_{\text{RA}}\right)={\dot{m}}_{a}c_{pa}\left(T_{\text{MA}}-T_{\text{RA}}\right) \end{array}$$

and the balance of the mass of water is expressed as

(2) $$\begin{array}{c} \left(1-\beta \right)\times {\dot{m}}_{a}\omega_{\text{RA}}+\beta \times {\dot{m}}_{a}\omega_{\text{OA}}={\dot{m}}_{a}\omega_{\text{MA}}\\ \beta \times {\dot{m}}_{a}\left(\omega_{\text{OA}}-\omega_{\text{RA}}\right)={\dot{m}}_{a}\left(\omega_{\text{MA}}-\omega_{\text{RA}}\right). \end{array}$$

#### 2.2.2. Cooling Coil Model

**Figure 4:**
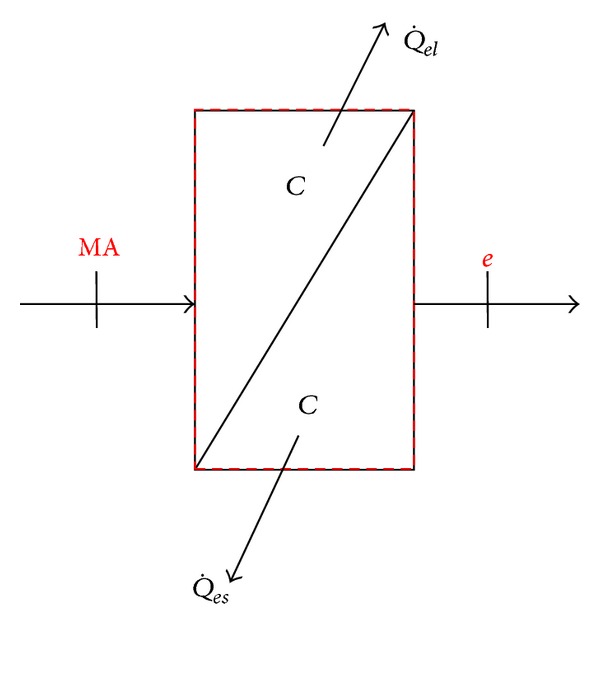
Schematic controlled volume of cooling coil model.

The energy in the air is transferred into the chilled water cycle, and the chilled water cycle delivers the energy to the evaporator. The evaporation of refrigerant in the evaporator takes the energy away, reducing the zone temperature and humidity. According to the air conditioning system, the chilled water temperature is generally 7~12°C. When the return air passes through the chilled water coil, if the chilled water temperature is lower than the return air dew-point temperature, the vapor in humid air is condensed for dehumidification. The mixed air enters the chilled water coil, taking the sensible heat (${\dot{Q}}_{es}$) and latent heat (${\dot{Q}}_{ls}$) of air away. The cooled and dehumidified air is fed to the heater. If the heat exchange rate is 100% when the air passes through the chilled water coil, it is steady flow process, and the moisture condensed on the coil is removed completely and immediately by gravity as shown in Figure 4.

Disregarding the variance in kinetic energy and potential energy, the energy conservation of chilled water is expressed as

(3) $$\begin{array}{c} -\left({\dot{Q}}_{es}+{\dot{Q}}_{el}\right)={\dot{m}}_{a}\left(h_{e}-h_{\text{MA}}\right). \end{array}$$

The humid air energy variation of (3) is divided into the energy of dry air and water vapor, expressed as

(4) $$\begin{array}{c} -\left({\dot{Q}}_{es}+{\dot{Q}}_{el}\right)={\dot{m}}_{a}c_{pa}\left(T_{e}-T_{m}\right)+{\dot{m}}_{a}h_{fg}\left(\omega_{e}-\omega_{m}\right). \end{array}$$

The sensible heat of air (${\dot{Q}}_{es}$) only influences the air temperature and the latent heat of air (${\dot{Q}}_{ls}$) only influences the air vapor, so (4) can be expressed as (5) and (6), respectively, as follows:

(5) $$\begin{array}{c} -{\dot{Q}}_{es}={\dot{m}}_{a}c_{pa}\left(T_{e}-T_{\text{MA}}\right) \end{array}$$

(6) $$\begin{array}{c} -{\dot{Q}}_{el}={\dot{m}}_{a}h_{fg}\left(\omega_{e}-\omega_{\text{MA}}\right). \end{array}$$

Equation (6) can be reduced to vapor mass conservation equation, expressed as

(7) $$\begin{array}{c} \frac{Q_{el}}{h_{fg}}={\dot{m}}_{a}\left(\omega_{e}-\omega_{\text{MA}}\right). \end{array}$$

#### 2.2.3. Heating Coil Model

**Figure 5:**
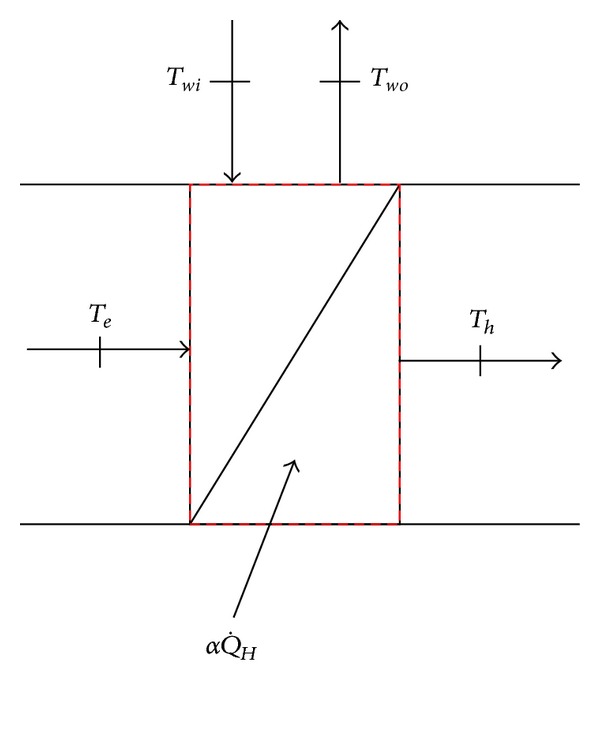
Schematic controlled volume of heating coil model.

It is switched on when the room temperature is lower than the set point. By heating the temperature of supplied air with heater, the heat energy is transferred to the air-conditioned zone, and let the temperature reach the set point. The heater is divided into hot-water heating, electric heater, and steam heating. In this study, the hot water coil is used as the heater of controlled temperature and humidity air conditioning system. The air through chilled water passes through the heating coil to take the heat into the air, and the heated air is fed to the humidifier as shown in Figure 5. The heating capacity of heater is pure sensible heat change, the heater output power ($\alpha {\dot{Q}}_{H}$) heats the air, and the humidity ratio of air-conditioned zone will not be influenced.

If the heating process is steady flow process, the energy conservation relationship of heater as shown in Figure 5 is expressed as

(8) $$\begin{array}{c} \alpha {\dot{Q}}_{H}={\dot{m}}_{a}c_{pa}\left(T_{h}-T_{e}\right)={\dot{m}}_{w}c_{pw}\left(T_{wi}-T_{wo}\right). \end{array}$$

#### 2.2.4. Humidifier Model

The humidifier output power ($\gamma {\dot{Q}}_{L}$) converts electric energy into latent heat, the moisture is added in the system by steam humidification so that the humidity of the air supply changes, and the system processed air is fed into the air-conditioned zone. The humidifier humidification is pure latent heat modification; therefore, only humidity changes, and the temperature of air-conditioned zone is not influenced. If the humidifier is steady flow process, it can be described by a simple vapor mass conservation equation as

(9) $$\begin{array}{c} \frac{\gamma {\dot{Q}}_{L}}{h_{fg}}={\dot{m}}_{a}\left(\omega_{s}-\omega_{h}\right). \end{array}$$

#### 2.2.5. Integration to a Zone Hygrothermal Model

In order to make the air-conditioned zone reach the set temperature and humidity, the controlled temperature and humidity air conditioning system must be used. The controlled temperature and humidity air conditioning system consists of chilled water coil, heater, and humidifier. The mathematical model of air-conditioned zone is simplified by the following assumptions.

1. The air-conditioned zone is an open and unsteady flow system in constant volume.
2. The air temperature and humidity in the air-conditioned zone are distributed uniformly.
3. The specific heat and density of air are constants.
4. There is no air leakage in the process.
5. The wall surface of air-conditioned zone is insulated.
6. The air is regarded as ideal gas.

According to thermodynamic energy conservation equation, the thermal model [17] can be expressed as

(10) Q˙C.V=W˙C.V.+∑out[m˙(h+V22+gz)] −∑in[m˙(h+V22+gz)]+dEC.V.dt.

If the kinetic energy and potential energy variations in the process are ignored and the system has not applied work, (10) is reduced to

(11) $$\begin{array}{c} {\dot{Q}}_{\text{C}.\text{V}}=\sum_{\text{out}}\dot{m}h-\sum_{\text{in}}\dot{m}h+\frac{dE_{\text{C}.\text{V}.}}{dt}. \end{array}$$

**Figure 6:**
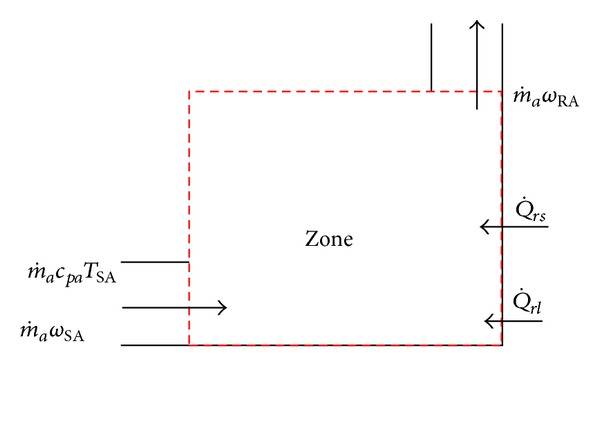
Schematic diagram of thermal model for air-conditioned zone.

Figure 6 shows that the air-conditioned zone state is significantly influenced by the persons, office equipment in operation, and various machine tools in the space and the heat transfer to the space is shown in Figure 2 so that the air state in the space changes. The temperature and humidity increase continuously, and the state keeps deviating from comfort zone. Different air conditioning loads influencing indoor state can be reduced to sensible heat load (${\dot{Q}}_{rs}$) and latent heat load (${\dot{Q}}_{rl}$). The energy conservation equation (12) for air-conditioned zone can be deduced from (11):

(12) $$\begin{array}{c} m_{\text{CV}}c_{v}\frac{dT_{\text{C}.\text{V}.}}{dt}={\dot{m}}_{a}c_{p}\left(T_{\text{OA}}-T_{\text{RA}}\right)+{\dot{Q}}_{rs}. \end{array}$$

According to the law of conservation of mass of vapor, the air-conditioned zone humidity change can be expressed as

(13) $$\begin{array}{c} m_{\text{C}.\text{V}}\times \frac{d\omega_{\text{C}.\text{V}}}{dt}={\dot{m}}_{a}\left(\omega_{\text{SA}}-\omega_{\text{RA}}\right)\times \frac{{\dot{Q}}_{rl}}{h_{fg}}, \end{array}$$

where ${\dot{m}}_{a}c_{p}(T_{\text{SA}}-T_{\text{RA}})$ and ${\dot{m}}_{a}(\omega_{\text{SA}}-\omega_{\text{RA}})$ are the energy and vapor mass, respectively, taken in or out of the system by various equipment types in the air conditioning cabinet, so (2) are combined with the thermal model of various equipment types of the air conditioning system in Section 2.2. The energy conservation and vapor mass conservation equations represent the indoor temperature and humidity variations, respectively, expressed as

(14) mcvdTC.V.dt=m˙acpa(Th−Te) +β×m˙acp(To−Tr)−m˙acp(Tm−Te)+Q˙rs

(15) mdωC.V.dt=m˙a(ωs−ωh) +βm˙a(ωo−ωr)−m˙a(ωm−ωe)+Q˙rlhfg.

## 3. Results and Discussion

In order to validate the controlled temperature and humidity air conditioning system model deduced in Section 2, MATLAB is used to create the dynamic simulation program of the system, and set the simulation condition working procedure of [6]. The changes in the temperature and humidity ratios of air-conditioned zone are compared with the literature, so as to validate the accuracy of the thermal model and the dynamic simulation program. When the dynamic simulation program is determined, a well-known control law is still required to direct the action of controlled temperature and humidity air conditioning system, so that the system function can be perfected. Therefore, the PID control is used and the cooling and dehumidification capability matching of chilled water is considered. Ziegler-Nichols rule is applied to obtain PID control parameters, so as to analyze the effectiveness of PID control of controlled temperature and humidity air conditioning system.

### 3.1. Modeling Validation

**Figure 7:**
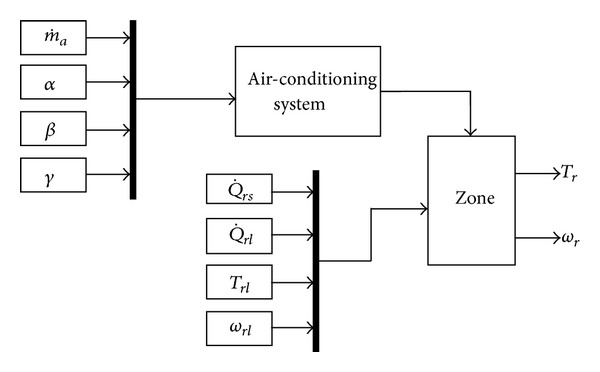
Block diagram of controlled temperature and humidity of an air conditioning system.

The dynamic simulation of the proposed model, based on the hypothesis in chapter 2, is built and processed by Matrix Laboratory (MATLAB) and is shown in Figure 7. The program simulates eight input parameters, which are zone sensible heat (${\dot{Q}}_{rs}$), zone latent heat (${\dot{Q}}_{rl}$), initial temperature of air-conditioned zone (*T*_*rl*_), zone initial humidity ratio (*ω*_*rl*_), air mass flow rate (${\dot{m}}_{a}$), heater capacity (*α*), outside air (*β*), and humidifier capacity (*γ*), respectively. When the above parameters are imported into the simulation, the program calculates the energy variation in the space resulting from various equipment types.

This study developed an open loop control test and set different air mass flow rates. The effectiveness of dynamic model of the system is validated by comparing the results of this study with the simulation results of the literature. This test neglected the external wall and heat transfer. The set conditions are shown in Table 1.

**Figure 8:**
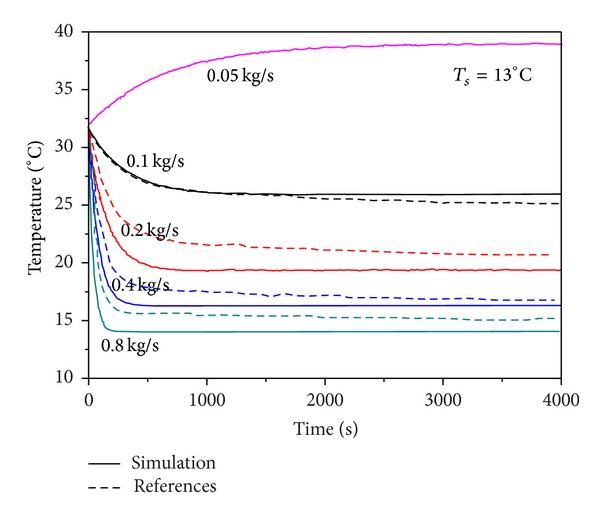
Zone temperature versus time for air mass flow rate compared with [6].

**Figure 9:**
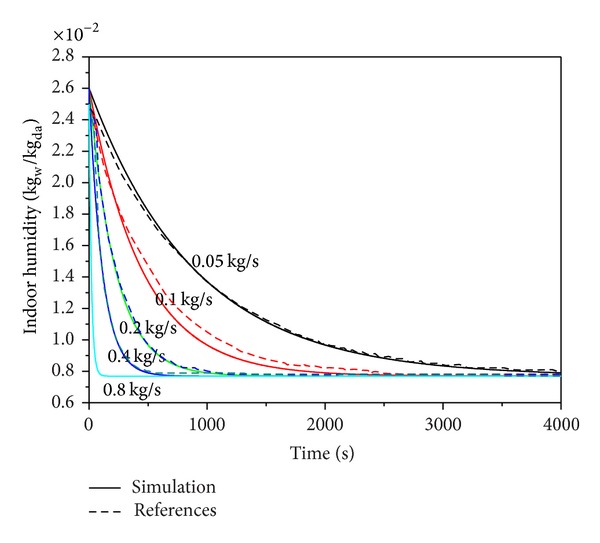
Zone humidity ratio versus time for air mass flow rate compared with [6].

The simulation results are shown in Figures 8 and 9. If there is only zone sensible heat, the stable temperature decreases as the air mass flow rate increases, and the humidity ratio converges to 0.00763 kg_w_/kg_da_. According to (14), when there is sensible heat load, the difference between zone temperature and chilled water temperature decreases with the indoor temperature. The temperature is stabilized until the heat taken away by the chilled water equals the sensible heat load. Therefore, with a larger air mass flow rate, the stable temperature is lower. When the system lacks air mass flow rate, the sensible heat load cannot be taken away adequately, so that the cooling is failed, and the return air temperature difference increases continuously. However, as the temperature difference changes, the heat load taken away by the chilled water increases and it reaches balance with sensible heat load at last. In terms of humidity ratio without latent heat, according to (5), regardless of ${\dot{m}}_{a}$, the outflow air humidity ratio is eventually converged. Relevant parameters are shown in Table 2. As seen, when ${\dot{m}}_{a}$ is large, the stabilization time is short. The simulation results are consistent with the trend in the literature. This study did not consider the wall heat transfer, and the simulation results were compared with the literature. At 4000 sec when stabilization was most approximately reached, the maximum error was 1°C, and the error rate was 4%. The humidity ratio converged to 0.763 kg_w_/kg_da_ with maximum error was 2.4%.

### 3.2. Simulation Results

PID control is a closed loop control system, commonly used in industry. The PID algorithm can provide a considerable control effect with good adjustment. Sensors are applied for detecting the present state of air-conditioned zone and returning them back to comparator. The state of air-conditioned zone reaches the set state through proportional control, integral control, and differential control. As it is simple and understandable and its adjustment does not need precise system model, it is widely used in various domains. The PID control input-output relationship is expressed as the following equation:

(16) $$\begin{array}{c} G_{o}\left(s\right)=\frac{U\left(s\right)}{E\left(s\right)}=K_{p}+\frac{S}{T_{i}}+T_{d}S, \end{array}$$

where *K*_*p*_, *T*_*i*_, and *T*_*d*_ are the proportionality, integral, and differential coefficients of PID controller. The proportional control regulates and controls the system according to the difference between input and output signals. However, the steady-state error cannot be removed by only proportional control. In order to eliminate steady-state error, the integral control is adopted. The integral term takes the integral of time and the integral term increases with time, so that the control output increases until the steady-state error is eliminated. In the process of regulation, the system may have oscillation even run out of control due to inertial or lag component, and the integral term magnifies the oscillation on the contrary. Therefore, the differential term is used to predict the variation trend to improve the interference effect. However, the three control parameters influence each other. Hence, how to obtain control parameters becomes an important but difficult problem in PID control.

The PID controller set-up parameters in this paper are designed according to Ziegler-Nichols empirical rule, which is regarded as a good parameter optimization in PID control [6]. The parameters are adjusted continuously to make system oscillation, so as to know the system response. The control parameter settings are derived from empirical equation, as shown in Table 3. In this paper, considering the matching of cooling and dehumidification effects of chilled water coil, the air mass flow rate (*m*_*a*_) is used as control parameter, and the heater and humidifier control is added. With the assistance of heater output power percentage (*α*) and humidifier output power percentage (*γ*), the loss of chilled water due to excessive cooling or dehumidification is compensated.

**Figure 10:**
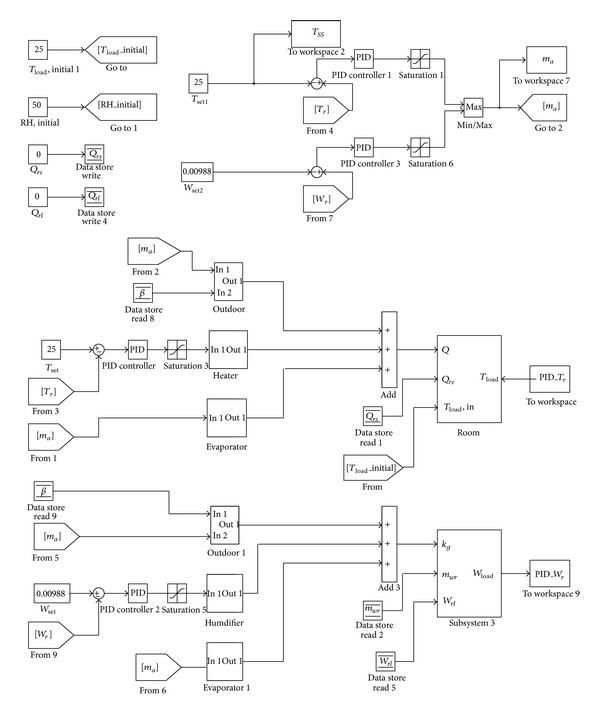
Schematic block diagram of PID control for the proposed system.

According to the system parameters of desired controlled temperature and humidity of air conditioning system, a PID controlled real time of the proposed air conditioning system can be built by Simulink as shown in Figure 10. Simulation begins with given initial conditions, and the controller adjusts P, I, and D, respectively, to control room temperature and humidity.

As shown in Table 3, the chilled water is equipped with two PID controllers, which feedback indoor temperature and humidity ratio data. Two air mass flow rate data are exported under control. As the dehumidification and cooling only depend on the chilled water coil, considering the system matching problem, this paper uses large value as the actual output. Therefore, if sensible heat or latent heat is removed excessively, the heater and humidifier are used for compensation.

This paper observes the convergence and stability of PID control based on constant temperature and humidity system thermal model in simulated summer environment. Moreover, it validates the feasibility of PID control in controlled temperature and humidity air conditioning system. The set conditions are described below.

1. *T*_set_ = 22°C; *ω*_set_ = 0.008 kg_w_/kg_da_.
2. *T*_*rl*_ = 32°C; *ω*_*rl*_ = 0.025 kg_w_/kg_da_.
3. Zone volume is 36 m^3^.
4. Sensible heat load is 1.3 kW.
5. Air mass flow rate is 0~0.3 kg/s.
6. Heater is 0~0.6 kW.
7. Humidifier is 0~1 kW.

**Figure 11:**
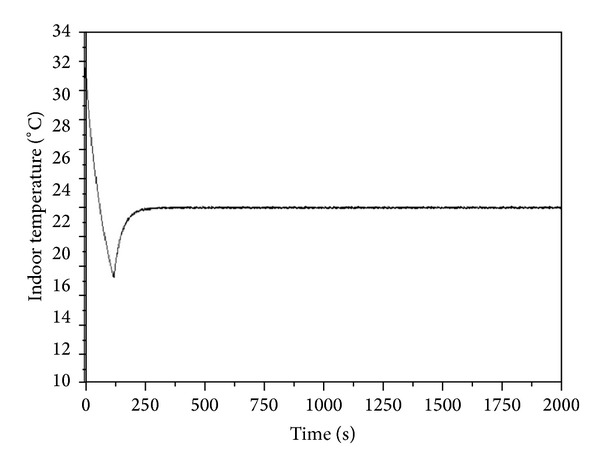
Zone temperature versus time for PID controller.

At the beginning, the overheat room temperature is detected by PID controller of chilled water coil, triggering the increase of mass flow rate to cool down the room temperature till reaching 17.81°C. The overcool temperature stimulates the PID controller of heater and the heater is working till setting temperature. The crossfunction of these two PID controllers results in a sharp control curve as shown in Figure 11.

**Figure 12:**
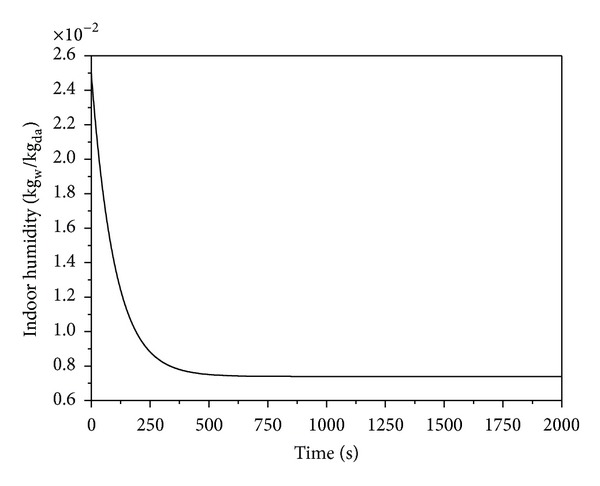
Zone humidity versus time for PID controller.

In humidifier, due to the fact the humidity ratio controlling needs only adjusting the chilled water coil, only one PID controller, which belong to chilled water coil, is working and results in a smooth curve as shown in Figure 12.

According to the indoor temperature and humidity ratio variations in Figures 6 and 7, under the PID control, the humidity ratio can be controlled well. However, in terms of temperature stability, the maximum disturbance is 0.14°C. Because there are two PID controllers, when the temperature and humidity approach the set values, and the output air between the two controllers, resulting in oscillation. In addition, as the air-conditioned zone cooling response speed is higher than dehumidification, when the temperature is reached, the chilled water still needs to continue dehumidification, so that the indoor temperature decreases to 17.81°C before it rises to the set value. The maximum overshoot of temperature is 4.19°C. The temperature and humidity are stable at 541 sec, but the steady state of humidity ratio is 0.0074 kg_w_/kg_da_, the steady-state error is 0.0006 kg_w_/kg_da_, and the error rate is 7.5%.

**Figure 13:**
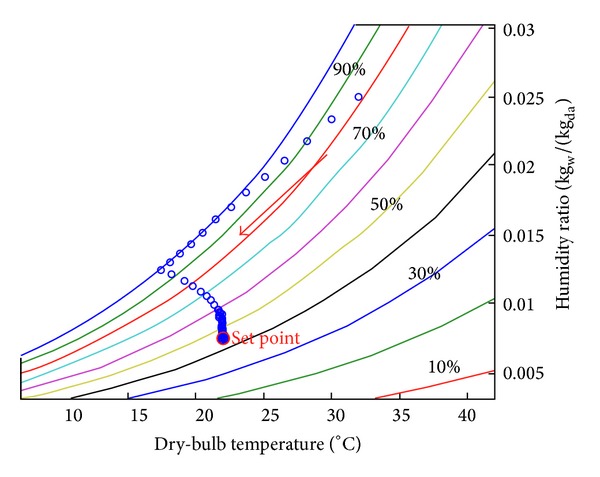
Zone air state change psychrometric chart.

The simulation data are displayed in the psychrometric chart, as shown in Figure 13. The air state changes in the air-conditioned zone in the PID control mode. First, it is high temperature and high humidity state and only the chilled water is used for cooling and dehumidification. Therefore, the air state almost develops towards the outflow air temperature and humidity point straightly. Soon after the temperature is lower than the set point, the heater is started up to lower the temperature to the set point. The matching of various equipment types stabilizes the air state inside the air-conditioned zone at the set point.

## 4. Conclusion

This study developed a thermal model for controlled temperature and humidity air conditioning system, including air-conditioned zone, air mixing stage, chilled water coil, heater, and humidifier. Based on the thermal model, MATLAB was used to simulate open loop control system, and it is compared with references to validate the feasibility of the system. As the wall heat transfer was disregarded, the maximum error between room temperature simulation and literature was 1°C, and the maximum error rate between humidity ratio simulation and the literature was only 2.4%. Finally, the PID controller was used for control. When the system matching was considered, the Ziegler-Nichols empirical rule was applied to control the air mass flow rate, humidifier output power, and heater output power. The results showed that the PID control could reach stabilization at 541 sec at high temperature and high humidity. The maximum overshoot was 4.19°C, the humidity ratio error rate was only 7.5%, and the maximum temperature disturbance was 0.14°C. The goal for constant temperature and humidity could be attained effectively.

## Figures and Tables

**Table 1:** Parameters of air-conditioned zone in [6].

Initial state of the zone	32°C DB	0.025 kg_w_/kg_da_
Zone volume (m^3^)	36	
Zone cooling load (kW)	Sensible heat	Latent heat
	1.3	0
Supply air temperature (°C)	13	

**Table 2:** Steady state of zone versus air mass flow rate.

Airflow (kg/s)	Temperature (°C)			Humidity ratio (kg_w_/kg_da_)		
	Reference [3]	Simulation	Error	Reference [3]	Simulation	Error
0.05				0.00781	0.00763	2.3%
0.1	24.95	25.95	−4%	0.00782	0.00763	2.4%
0.2	20.28	19.47	3.9%	0.00782	0.00763	2.4%
0.4	17.01	16.23	4.5%	0.00775	0.00763	1.5%
0.8	15.16	14.62	3.5%			

**Table 3:** Parameters of PID controllers.

Control parameters	Cooling coil		Heater	Humidifier
	Airflow (*m*_*a*_)		Percentage of output power (*α*)	Percentage of output power (*γ*)
Feedback parameters	Zone temperature	Zone humidity ratio	Zone temperature	Zone humidity ratio
*K*_*p*_	14.7	52.1	34.7	28.2
*K*_*i*_	1	1	1	1
*K*_*d*_	0.25	0.25	0.25	0.25

## Nomenclature

${\dot{m}}_{a}$: Mass flow rate of the air stream (kg/s)
*c*_*p*_: Specific heat (kJ/kg-°C)
*T*: Temperature (°C)
*ω*: Humidity ratio (kg_w_/kg_da_)
*h*: Enthalpy (kJ/kg)
*h*_*e*_: Enthalpy of air through chilled water (kJ/kg)
*h*_*fg*_: Latent heat of vaporization of vapor (kJ/kg)
${\dot{Q}}_{H}$: Maximum input power of heater (kJ)
${\dot{Q}}_{L}$: Maximum output power of humidifier (kJ).

### Subscripts

*a*: Air
da: Dry air
*e*: Air through chilled water
*h*: Heater
*l*: Latent heat
*s*: Sensible heat
*w*: Water
MA: Mixing air
OA: Outside air
RA: Return air
WO: Return water
Wi: Supply water.

### Greek Letters

*α*: Heater input power percentage
*β*: Outside air damper opening
*γ*: Humidifier output power percentage.
